# A novel and easy technique for restoring grossly decayed mandibular molar with a modified cast-post

**DOI:** 10.11604/pamj.2018.31.129.14394

**Published:** 2018-10-22

**Authors:** Tej Jitesh Joshi, Gaurang Mistry, Devanand Shetty, Omkar Shetty, Sahil Singh, Shraddha Rumde

**Affiliations:** 1 Department of Prosthodontics, DY Patil School of Dentistry, Nerul, Navi-Mumbai, India; 2 Department of Periodontics, DY Patil School of Dentistry, Nerul, Navi-Mumbai, India; 3 DY Patil School of Dentistry, Nerul, Navi-Mumbai, India

**Keywords:** Cast post, sliding pre-fabricated post, zirconia

## Abstract

The article addresses the difficulty of restoring a grossly carious molar tooth, where very little tooth structure is left after caries excavation. When enough sound tooth structure is missing for satisfying the ferrule effect, a clinician can follow this technique and easily restore such teeth. A step by step procedure, including instrumentation and materials and a new impression technique, is described in detail, with clinical photographs. This technique results in the fabrication of a robust and extremely retentive post and core on which to place fixed prosthodontic restorations. A 2 and half year study showed that the tooth well in function with no signs of any problem. The author has restored around many decayed molars using this technique. Over a period of three years, no failure was reported. With an increasing demand on the dentist for restoring a structurally compromised teeth, this technique provides the patients with a robust prosthodontic solution.

## Introduction

The definition of prosthodontics is, "Prosthodontics is the dental specialty pertaining to the diagnosis, treatment planning, rehabilitation, and maintenance of the oral function, comfort, appearance, and health of patients with clinical conditions associated with missing or deficient teeth and/or maxillofacial tissues by using biocompatible substitutes." The restoration of a grossly carious molar tooth is always questionable. With the advent of Implants, it is always a tough choice to make. But still, there are many people who cannot afford an implant treatment. Also there are many limitations when it comes to implant surgeries. So it should always be a dentists concern to "preserve what's remaining". According to the author, this should be the first choice of treatment before the dentists seek implants as their first treatment option in such cases. Also the proprioceptive response is not lost as the PDL is preserved for that tooth. A dentist with good knowledge of prosthodontics will never deny the importance of having proprioceptive response. Taking all these factors into consideration, this is one of the best choice for restoring badly broken down molar teeth. A comprehensive literature review has been presented by Chiche *et al* [1]. This traced back to 1911 where one of the first methods was suggested by Robin. In the same article Chiche et al described his own modality, where a prefabricated plastic post was inserted into the palatal canal of a maxillary molar and the impression of the remaining tooth, already prepared for a crown, was taken with the plastic post in it. A crown was then fabricated and a gold post cast from a plastic post was inserted through the crown immediately following crown cementation. Mora and Firtell [1] suggested replacing a graphite post in the second canal with a metal post, Weiner in 1981. Chand developed a post selection guide for the para post system (Whaledent International, USA) for a pre-operative assessment of the para post drill suitable for post hole preparation. He also addressed the problem of post hole clearing before cementation. Sadan *et al* [1] suggested a similar technique to Chiche's but for indirect fabrication of two separate castings, first-the post and then-the core for mandibular molars. After cementation of this assembly a crown was manufactured from a new impression. Most clinicians suggested, if possible, preparation of the dowel space to within 3-5mm of the apical seal. This is not considered to be a requirement these days as a post length equal to the length of the expected crown is thought to be sufficient. However, in the author's opinion, in divergent roots, when two posts are utilized even this length is not necessary. Neither is it required to achieve a diameter of the post equal to one-third of the root width as has been suggested by Johnson *et al* [1]. Trabert and Coone [1]. Indeed, as the posts are divergent, much shorter ones should provide adequate retention. The luting cement has been changed as the new resin reinforced glass ionomer cement is claimed by the manufacturer to adhere chemically to dentine and should provide better retention than zinc phosphate. In the technique described in this paper, isolation of badly broken down molars was achieved by rubber dam.

## Patient and observation

**Figure 1:**
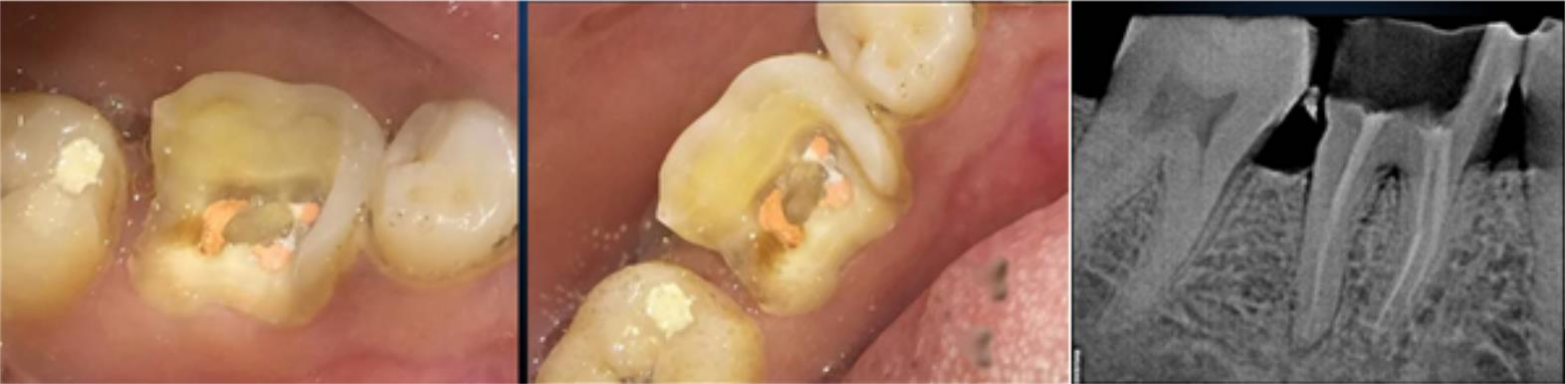
Pre operative, clinical picture and IOPA

On 27^th^ of august, 2015, a 37 year old male patient reported to the Department of Prosthodontics, DY Patil School of Dentistry, Navi-Mumbai. His chief complaint was of a fractured tooth which had undergone a root canal treatment a month back. The tooth was evaluated to rule out vertical fracture using trans-illumination and gentian violet dye. No fractures were detected Figure 1.

**Figure 2:**
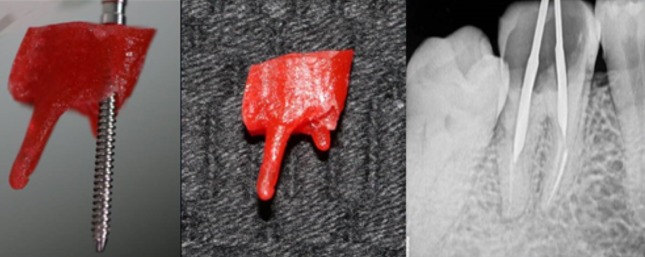
Cast-post direct resin pattern and IOPA with post space preparation

**Figure 3:**
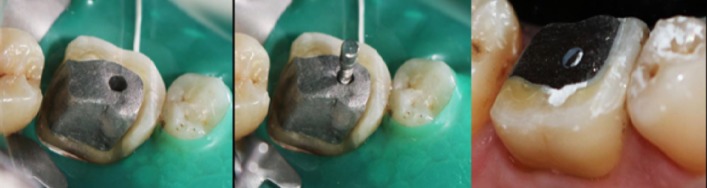
Try in of cast post and Pre-fabricated post; and immediately after cementation

**Cast-post procedure**: The lingual aspect of the tooth was a millimeter sub-gingival. So a crown lengthening procedure was carried out using scalpel. There was adequate keratinized tissue present on the lingual aspect after crown lengthening procedure. No osseous reduction was required [2]. Two weeks after crown lengthening, the canals were prepared using no. 2 and no. 3 peso-reamers in mesio-buccal and distal canal respectively. A direct pattern was made using GC Pattern Resin and a Prefabricated no. 2 MANI Post. The pattern was made with the prefabricated post in the mesio-buccal canal. After the resin has set, the prefabricated post was rotated and removed from the resin post and core. The resin pattern was carefully removed from the distal canal after it had set completely. The pattern was casted using semi-precious alloy. Both, the pre-fabricated post and the finished cast past was sand blasted using 50 microns aluminum oxide. Also a small slot was prepared on the prefabricated post at the occlusal level of the cast-core. This was done to felicitate easy removal of the occlusal excess without using any rotary tools, as the vibrations caused by the rotary instruments to cut off the excess pre-fabricated post, may loosen the bond of the unset cement Figure 2, Figure 3. The tooth was isolated using a rubber dam. The tooth was treated using 37% phosphoric acid. The canals were rinsed and dried using distilled water and paper points. The cement used was resin modified GIC [3]. The cement was dispensed using dispensing tips into both the prepared canals. The cast post was coated with some more cement and seated into the tooth. The pre-fabricated post was also immediately pushed into the mesio-buccal canal before the cement had passed its working time. The cement was allowed to set for 5 minutes. After the initial set had occurred, the excess part of the prefabricated post was tilted and broken off from the assembly. The slot made at the occlusal level provided an easy means to cut the excess off and allowed the patient to occlude.

**Figure 4:**
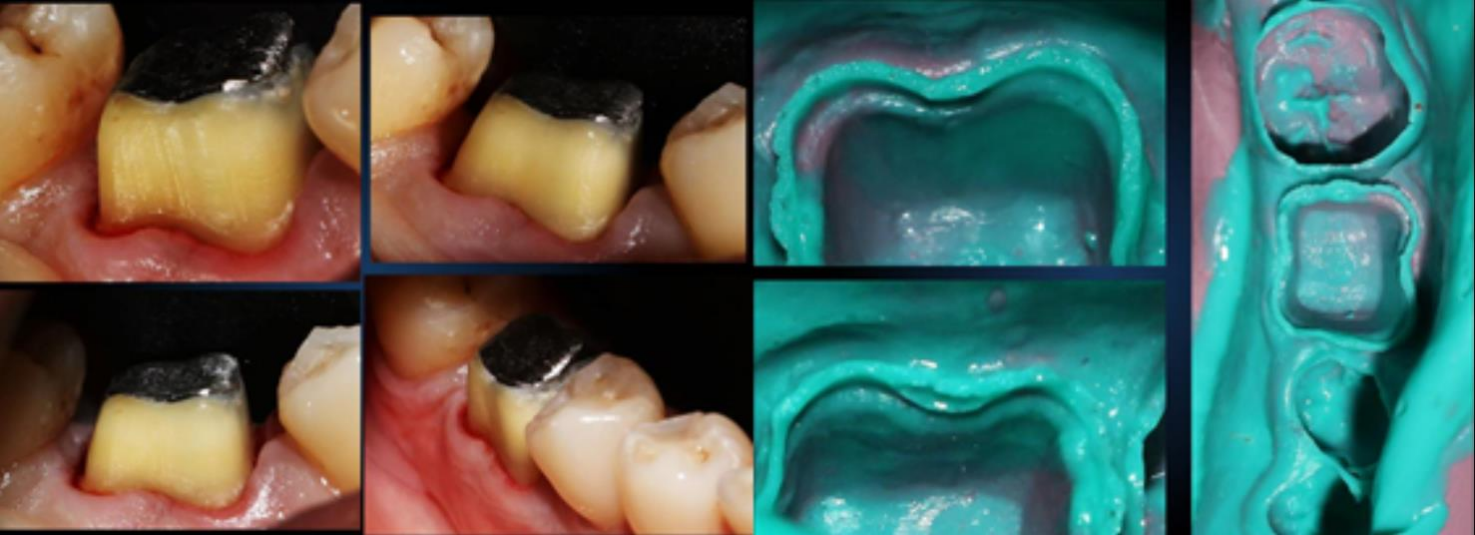
Tooth prep and addition silicone impression

**Figure 5:**
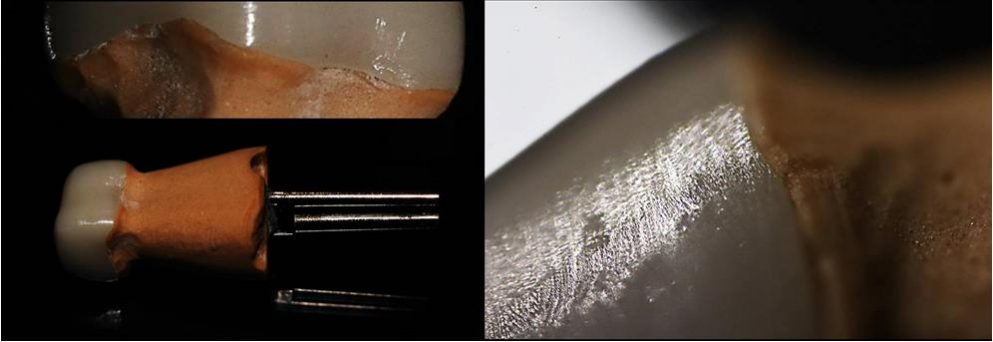
Zirconia coping depicting excellent marginal fit

**Figure 6:**
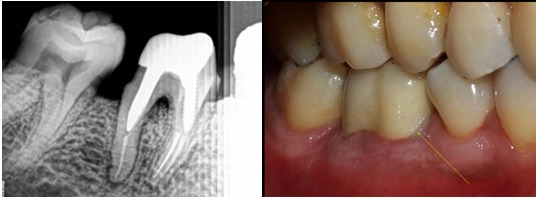
Post op IOPA and immediately post cementation

**Tooth preparation procedure**: Post cementation, after 48 hours, the patient was recalled. Tooth preparation was done during this appointment. The margins chosen were chamfer on distal, lingual and buccal aspect Figure 4, Figure 5. A knife edge margin was chosen on the mesial aspect. Studies claim that the zirconia preparation with no margin and a sub-gingival margin has a very good longitivity [4]. Modern day zirconia allows the clinician to have margins as thin as 0.2 mm. Also the highly polished zirconia facilitates hemi-desmosomal attachment with the gingiva. This also prevents marginal leakage Figure 6.

## Discussion

**Figure 7:**
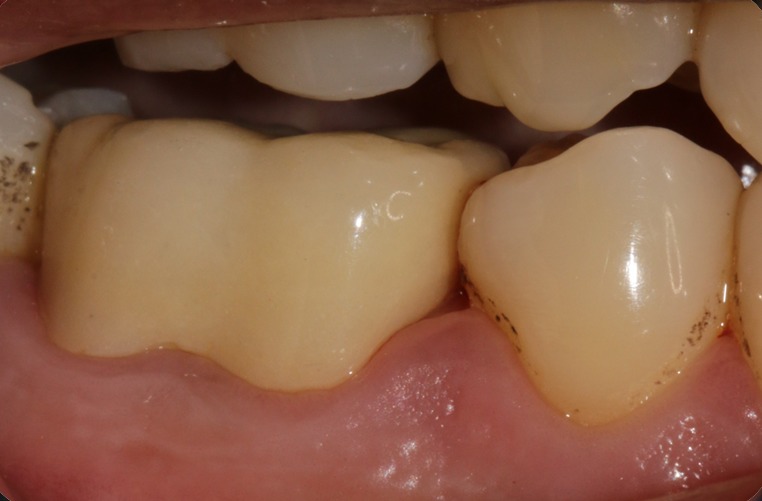
Thirty months follow-up

This procedure is very predictable and easy to perform. E. V. Bass, in his article, has quoted that "The author has placed over 600 restorations of this type over a five year period, without any reported failures." Even in this case, a two and half year follow-up provided that the prognosis of the tooth restored with this kind of restoration was very good. Fiber-posts are used quite often these days. Manufacturers claim that the fiber posts have elastic modulus similar to that of dentin. But the practitioners fail to understand that the geometry, length of the post and diameter of the post, as well as that of the residual dentin, plays a vital role in deciding the elasticity. The use of passive fit, metal posts have given time tested results, and are best to use according to the author. The use of resin modified glass ionomer cement was questioned for cementation of cast posts, as isolation was a problem. But the use of rubber dam solved the issue. The resin modified GIC from kerr, was chosen for this purpose as its properties were better as compared to other available luting agents. Also the ease of dispensing, and the availability of micro-tip, made it possible to avoid any voids in the canal space while cementing the posts. In their article, by R. Magallanes Ramos and P. Venuti, a paradigm shift was made on the approach on finish lines. In their article, they claim that a shoulder-less approach preserves a lot of natural tooth structure as compared to that of a shoulder or chamfer margins. Also a good ferrule effect is obtained by this technique, which increases the longevity of the restoration [5], Figure 7.

## Conclusion

In this article, a new and modified technique is described for restoration of mandibular molars after endodontic treatment, where very little is left of the clinical crown. Especially in divergent roots, using easily available instruments, a predictable restorative procedure can be easily performed. A new impression technique for cast post and core restoration, a new luting technique and a new technique for tooth preparation has been described in the article by the author.

## Competing interests

The authors declare no competing interests.
